# Histone Deacetylase Inhibitor-Induced CDKN2B and CDKN2D Contribute to G2/M Cell Cycle Arrest Incurred by Oxidative Stress in Hepatocellular Carcinoma Cells via Forkhead Box M1 Suppression

**DOI:** 10.7150/jca.60027

**Published:** 2021-06-22

**Authors:** Hae-Ahm Lee, Ki-Back Chu, Eun-Kyung Moon, Fu-Shi Quan

**Affiliations:** 1 Medical Research Center for Bioreaction to Reactive Oxygen Species and Biomedical Science Institute, School of Medicine, Graduate School, Kyung Hee University, Seoul, Republic of Korea.; 2 Department of Biomedical Science, Graduate School, Kyung Hee University, Seoul, Republic of Korea.; 3 Department of Medical Zoology, School of Medicine, Kyung Hee University, Seoul, Republic of Korea.

**Keywords:** Oxidative stress, HDACi, cell cycle arrest, cyclin-dependent kinase 4/6, FOXM1

## Abstract

Forkhead box protein M1 (FOXM1) is a pivotal regulator of G2/M cell cycle progression in many types of cancer. Previously, our study demonstrated that histone deacetylase inhibition (HDACi) sensitizes hepatocellular carcinoma cells (HCC) to oxidative stress through FOXM1 suppression. However, the mechanism underlying its suppression by HDACi still requires elucidation. We hypothesized that HDACi induce genes responsible for destabilizing and inactivating FOXM1. The transcriptome in the HepG2 was revealed by massive analysis of cDNA end (MACE). Expression of mRNA and proteins were analyzed by quantitative real-time PCR (qPCR) and western blot, respectively. Cell cycle was analyzed by fluorescence-activated cell sorting (FACS). Oxidative stress and HDACi suppressed CDK4/6 levels while enhancing CDK inhibitor 2B and 2D (CDKN2B and CDKN2D) expressions in HCC. Palbociclib, a specific inhibitor of CDK4/6, induced G2/M cell cycle arrest in HCC by down-regulating phosphorylation level of FOXM1, and its downstream target genes such as aurora kinase A (AURKA) and polo-like kinase 1 (PLK1). HDACi treatment increased the ubiquitination level of FOXM1 by suppressing ubiquitin-specific peptidase 21 (USP21), which deubiquitinates FOXM1. Inhibiting FOXM1 degradation with MG132 treatment affected neither palbociclib-induced G2/M cell cycle arrest nor expression of its target genes. Double knockdown of CDKN2B and CDKN2D reduced the oxidative stress and HDACi-induced G/2M cell cycle arrest. In conclusion, oxidative stress and HDACi synergistically cause G2/M cell cycle arrest via CDKN2 induction, which sequentially inhibits CDK4/6, FOXM1, and its downstream target genes AURKA, PLK1, and CCNB1 phosphorylation in HCC.

## Introduction

Human hepatocellular carcinoma (HCC) is one of the most common cancers and global mortality associated with it has increased during the past 15 years, primarily due to delayed detection and frequent recurrence that hinders HCC therapy 1. Many risk factors for HCC have been well-established, which include hepatitis B and C viruses, alcohol abuse, non-alcoholic fatty liver disease, obesity, diabetes, and iron accumulation 2. Whole exome sequencing results from HCC have delineated genetic mutations in oncogenes and tumor suppressor genes which were attributed to specific risk factors 3. Reactive oxygen species (ROS) are a well-known inducer of DNA damage and a carcinogenic factor, thus many researchers have tried to modulate ROS levels in cancer for its prevention and therapy 4. Higher than normal intracellular ROS production can be detected in cancer cells as a consequence of genetic, metabolic, and tumor microenvironmental changes 5. The vast quantities of accumulated ROS generate numerous oncogenic mutations that subsequently lead to tumor survival, progression, and development of drug resistance 6. To offset this imbalance, antioxidant activity is increased in the tumor cells to maintain redox homeostasis. However, this altered redox balance leaves the cancer cells more vulnerable to ROS accumulation in comparison to their normal counterpart 7. Therefore, despite the diverse side effects, ROS-producing anticancer drugs have been developed and are currently used in clinical settings for cancer therapy 8.

Forkhead box protein M1 (FOXM1) belongs to the forkhead box transcription factor family. Unlike FOXO, another forkhead box transcription factor that is activated in quiescent cells and inhibits cell proliferation, FOXM1 is expressed in proliferating cells and acts as a master regulator of cell cycle progression 9, 10. Additionally, FOXM1 plays a critical role in other biological processes such as cell differentiation, DNA damage repair, tissue homeostasis, angiogenesis, and apoptosis 11. In cancer biology, FOXM1 is an important oncogene which is involved in tumor initiation, invasion, metastasis, and angiogenesis 12. Overexpression of FOXM1 has been reported in diverse malignancies, including but not limited to cancers of the prostate, breast, lung, ovary, colon, pancreas, stomach, bladder, liver, and kidney 13. For this reason, FOXM1 has been established as a potential target in human cancer therapy 14, 15.

Histone deacetylase inhibitors (HDACi) emerged as anticancer drugs decades ago 16. HDAC removes acetyl groups from the lysine residue in the histone tails, which results in transcriptionally inactive heterochromatin. Though numerous tumor suppressor genes are inactivated in cancer cells through epigenetic alterations, HDACi treatment can restore the expression of these genes and ultimately result in cell cycle arrest or apoptosis 17. The United States Food and Drug Administration (FDA) has approved HDACi such as vorinostat, belinostat, panobinostat for treatment of hematologic malignancies. Additionally, several other HDACi including the panHDACi (givinostat, resminostat, bexinostat, and quisinostat) and class-specific selective HDACi (CHR-2996 for class I and rocilinostat for class II) are currently undergoing clinical trials 18. As with other chemotherapeutic agents, HDACi-resistant cancers have been reported and multitudes of studies are investigating the therapeutic effects of anticancer drugs targeting DNA repair pathways, proteosome inhibitors, hormones, and tyrosine kinase inhibitors combined with HDACi 19.

Our previous study has demonstrated that treatment with histone deacetylase inhibitor (HDACi) suppressed FOXM1 expression and sensitized HCC cells to oxidative stress, which led to G2/M cell cycle arrest at a low level of oxidative stress 20. However, the mechanism underlying the suppression of FOXM1 by HDACi was not elucidated. To the best of our knowledge, HDACi and oxidative stress-mediated suppression of FOXM1 and its downstream pathway via CDKN2 induction has not been reported to date. The present study demonstrated that oxidative stress and HDACi induced expression of inhibitor of cyclin-dependent kinase 2 (CDKN2) family which inhibit the kinase activity of CDK4/6, subsequently resulted in decreased phosphorylation level of FOXM1 and expression of its target genes in HCC.

## Materials and Methods

### Cell culture and viability assay

Human hepatocellular carcinoma cell lines (HepG2 and Hep3B) were cultured in Dulbecco's Modified Eagle's Medium (DMEM) supplemented with 10% fetal bovine serum (FBS) and 1% penicillin/streptomycin at 37 °C with 5% CO_2_. Cell viability was analyzed using Cell Counting Kit 8 (CCK8). Cells (5 x 10^3^/well) were seeded in 96 well plate with 100 μl medium. After 24 h, cells were treated with agents. The next day, 10 ul of CCK 8 solution was added to the wells and then incubated for 2 h. Optical density was measured with a microplate reader at 450 nm (EZ Read 400, Biochrom Ltd., Cambridge, UK). For knockdown, siRNA (final 1 μmol/L) was transfected using the INTERFERin^®^ transfection reagent (Polyplus, Illkirch, France) following the manufacturer's instructions.

### Antibodies and agents

Antibodies to detect human CDK4, CDK6, CDKN2B, CDKN2D, AURKA, PLK1, CCNB1, and phospho-CCNB1 (pSer147) were purchased from Cusabio (Hubei, China). Ubiquitin, FOXM1 (ChIP grade), and p-FOXM1 (pThr600) antibodies were purchased from Santa Cruz Biotechnology, Inc (Dallas, TX, USA). CDKN2B and CDKN2D siRNA were purchased from Santa Cruz Biotechnology, Inc (Dallas, TX, USA). MS-275, suberanilohydroxamic acid (SAHA), palbociclib, tertiary-butylhydroperoxide (tBHP), and MG132 were purchased from Tocris Bioscience (Bristol, UK).

### RNA extraction and RNAseq

Total RNA was extracted using RNAeasy mini kit (Qiagen, Venlo, Netherlands). RNA quality and quantity were determined using NanoDrop One (Thermo Fisher Scientific, Waltham, MA, USA). Massive Analysis of cDNA End (MACE), an RNAseq method was performed as described previously 20. Briefly, cDNA library was constructed using QuantSeq 3' mRNA-Seq Library Prep Kit (Lexogen, Inc., Vienna, Austria). NextSeq 500 (Illumina, Inc., San Diego, CA, USA) was used to perform high-throughput sequencing. QuantSeq 3' mRNA-Seq reads were aligned using Bowtie2. Bowtie2 indices were generated from either genome assembly sequence or the representative transcript sequences for aligning to the genome and transcriptome. Normalized read count (NRC) was calculated using Edge R within R (R development core team, 2016). Analysis of gene ontology (GO) and GO annotations were performed using the quickGO database (https://www.ebi.ac.uk/QuickGO/). Heatmap of differentially expressed genes was generated with Multiple Experiment Viewer (MeV) software. Fastq files from MACE were deposited in the NCBI SRA repository under accession number PRJNA678827 (https://www.ncbi.nlm.nih.gov/Traces/study/?acc=PRJNA678827).

### Quantitative real-time polymerase chain reaction (qPCR)

Total RNA extraction and determining RNA quality and quantity were performed as described above. Two micrograms of total RNA was used to synthesize cDNA. RevertAid^TM^ First Strand cDNA synthesis kit (Fermentas, Vilnius, Lithuania) was used to synthesize single strand cDNA according to the manufacturer's recommendation. Then qPCR was perform using micPCR (PhileKorea, Seoul, Korea) as described previously 20. Critical threshold (Ct) values were normalized to *Gapdh* and used to calculate ΔCt values. Data were presented as fold change of 2^-ΔCt^. All primer sets used in the present study are shown in Table S1.

### Fluorescent activated cell sorting (FACS)

FACS analysis for cell cycle arrest was conducted as described previously 20. Briefly, collected cells were washed with PBS two times followed by ethanol fixation at 4 °C for 2 h. After fixation, cells were washed with PBS two times and incubated with RNase A (10 ug/ml) at 37 °C for 30 min. The cells were stained with propidium iodide (PI, 10 ug/ml) for 30 min in the dark. Cell cycle analysis was performed using Accuri C6 (BD Biosciences, CA, USA).

### Immunoprecipitation (IP) and western blotting

Cells (5 × 10^5^) were seeded in 60 mm dishes and the next day, cells were treated with drugs for 24 h. After washing with PBS, cells were lysed with lysis buffer. For IP, cells were lysed with non-denaturing lysis buffer [20 mmol/L Tris-HCl (pH 8), 137 mmol/L NaCl, 10% Glycerol, 1% NP-40, and 2 mmol/L EDTA] containing protease inhibitor cocktail. Cell lysates were incubated with primary antibody (2 ug) at 4 °C overnight (O/N). Target protein-antibody complex was precipitated using protein A/G agarose (60 ul of 50% slurry) at 4 °C for 2 h. The immune complexes were washed with lysis buffer three times. After elution of the immune complex, a western blot was performed to detect precipitated target protein. For western blotting, cell lysates (30 ug) were separated on a polyacrylamide gel and transferred to a nitrocellulose (NC) membrane. Transferred membrane was incubated with 5% skim milk in TBST buffer (25 mmol/L Tris base, 150 mmol/L NaCl, and 0.1% tween 20) for 1 h. The membranes were incubated with primary antibody (0.1~0.5 ug/ml) at 4 °C, O/N. After washing the membrane with TBST three times, secondary antibodies (1:5000) were added and incubated for 1 h. Target protein bands were developed using enhanced chemiluminescence (ECL) agent (Thermo Fisher Scientific, Waltham, MA, US) in the darkroom. The band density was calculated with ImageJ software.

### Chromatin immunoprecipitation (ChIP)

ChIP assay was performed using the EpiTect ChIP OneDay Kit (Qiagen, Venlo, Netherlands) according to the manufacturer's instructions. Cells were fixed with fresh formaldehyde (1% in PBS) for 5 min. Fixation was quenched with 125 mmol/L glycine for 10 min. Cells were lysed with SDS lysis buffer containing protease inhibitor cocktail. Soluble chromatin was generated by sonication (60 amp, 15 cycles of 10 sec sonication, and 50 sec cooling in the ice). After pre-clearing with protein A/G agarose, ChIP grade anti-FOXM1 antibody (2 μg) was added and incubated at 4 °C for O/N. The antibody-chromatin complex was precipitated with protein A/G agarose and then sequentially washed with a low-salt solution, high-salt solution, LiCl solution, and Tris-EDTA solution twice. Precipitated chromatin was eluted with elution buffer (1% SDS and 0.1 mol/L NaHCO_3_), followed by protein lysis with protease K at 45 °C for 30 min. DNA was eluted with DNA binding columns. FOXM1 binding on the promoter region of target genes was analyzed using qPCR. Primer sequences for ChIP assay are shown in Table S1.

### Statistics

Results are expressed as mean±SD. Kruskal-Wallis test and 1-way ANOVA followed by Dunnett's post hoc comparison test were used for data analysis; differences were considered significant at *p*<0.05. The Student-*t* test or χ^2^ test were applied for analyzing significant differences between the 2 groups.

## Results

### HDACi suppressed the expression of Cdk 4 and 6 while inducing the expressions of CDK4/6 inhibitors

**Figure 1:**
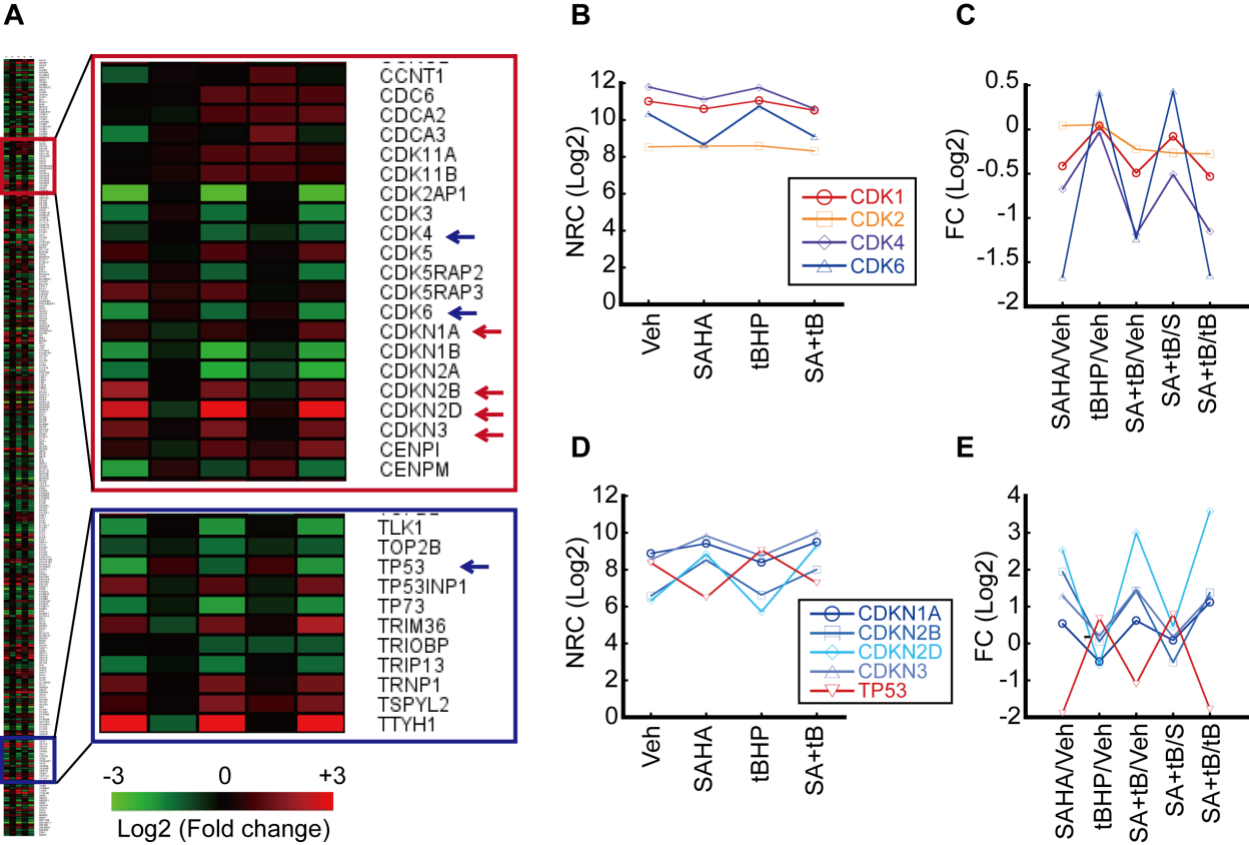
** Analysis of transcriptome related to the cell cycle.** HepG2 cells were treated with vehicle, tBHP (25 µmole/L), SAHA (1 µmol/L), or tBHP+SAHA. Transcriptome was analyzed by MACE, an RNAseq. **A.** Heatmap of gene expressions which changed more than 2-fold and are included under the GO Term “cell cycle”. **B.** Normalized read count (NRC) of *Cdk1*, *2*, *4*, and *6*. **C.** Fold change (FC) of *Cdk1*, *2*, *4*, and *6*. FC of *Cdk1* and *2* were barely changed by tBHP, SAHA, or combinatorial treatment with tBHP and SAHA. *Cdk4* and *6* FC were dramatically decreased by SAHA treatment. **D.** NRC of *Cdkn1a*, *2b*, *2d*, *3*, and *Tp53*. **E.** FC of *Cdkn1a*, *2b*, *2d*, *Cdkn3*, and *Tp53*. The expression of *Cdkn2b* and* Cdkn2d* was increased whereas that of *Tp53* was decreased by SAHA treatment.

Our previous study has shown that HDACi sensitizes HCC cells to oxidative stress and induces G2/M cell cycle arrest 20. Thus, we analyzed genes related to the cell cycle including cyclin-dependent kinase (CDK) and inhibitor of CDK (CDKN). Genes categorized under the GO Term “cell cycle” that underwent more than 2-fold changes upon treatment with *tert*-butylhydroperoxide (tBHP), suberanilohydroxamic acid (SAHA), or tBHP and SAHA combined (SA+tB) were used to generate a heatmap (Fig. 1A). Normalized read count (NRC) showed that CDK1 and 2 were changed negligibly by tBHP, SAHA, or combined treatment. However, NRC of Cdk4 and 6 were decreased by SAHA or combined treatment with SA+tB (Fig. 1B). Similarly, the fold change (FC) of NRC showed dramatically decreased Cdk4 and 6 upon SAHA or SA+tB treatments (Fig. 1C). Contrary to this, the NRC of CDK inhibitors were increased by SAHA or SA+tB treatments (Fig. 1D). Consistent with this finding, the FC of CDKN2B and CDKN2D were dramatically increased through SAHA or combined treatment with SA+tB except for Tp53 (Fig. 1E).

### Oxidative stress and HDACi suppressed CDK4 and 6 expressions, whereas the expressions of CDK4/6 inhibitors were enhanced in HCC

**Figure 2:**
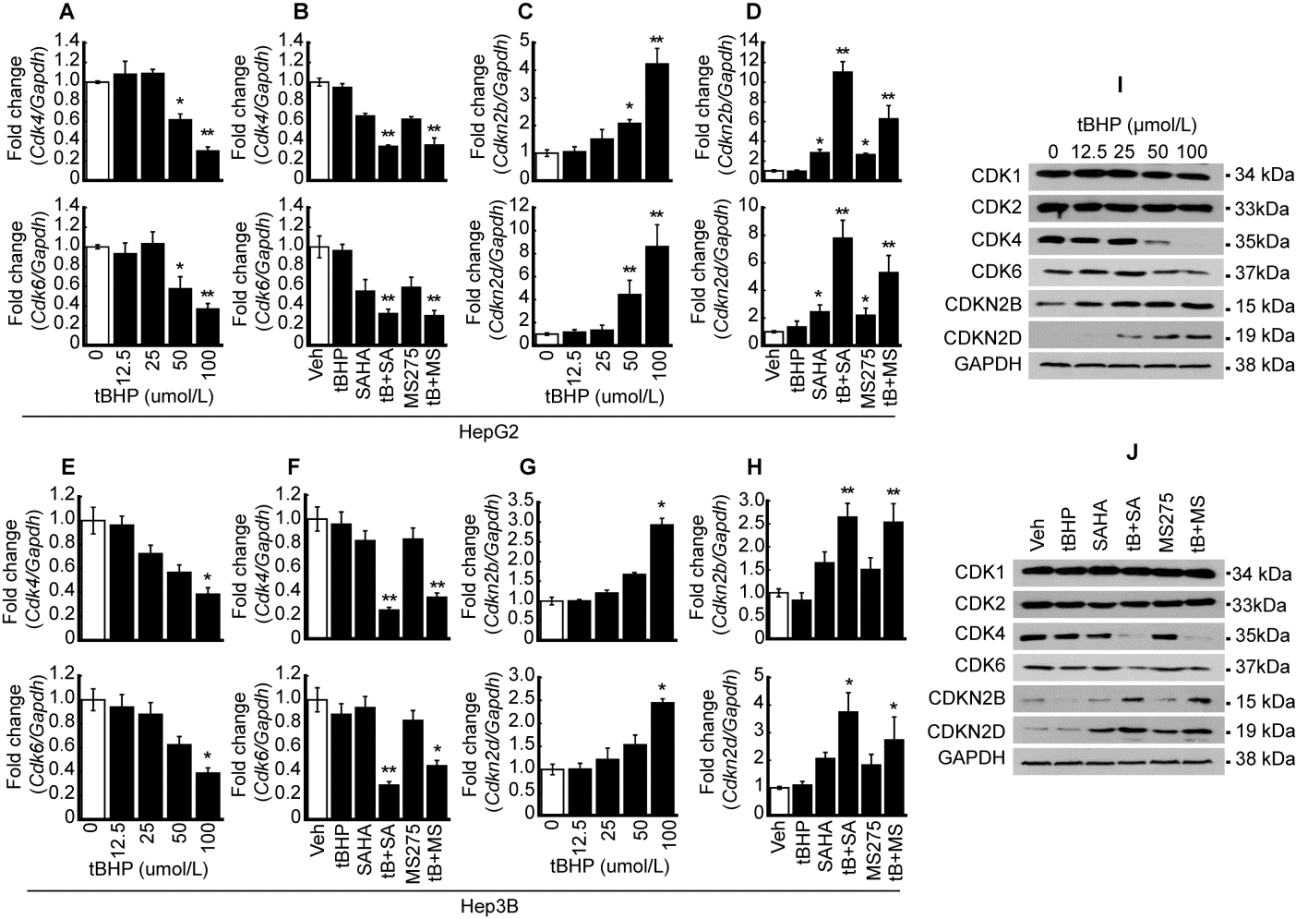
** Synergistic effect of oxidative stress and HDACi on the expression of CDK and CDKN.** To validate MACE results, oxidative stress-induced HCC cells (HepG2 and Hep3B) were used to perform qPCR and western blot. **A.** mRNA expressions of *Cdk4* and *6* were analyzed by qPCR in HepG2 cells. Expression of *Cdk4/6* was significantly decreased by oxidative stress induced by tBHP. **B.** HepG2 cells were treated with vehicle, tBHP (25 µmol/L), SAHA (1 µmol/L), MS-275 (1 µmol/L), or combination with tBHP and HDACi. Expressions of *Cdk4* and* 6* were slightly decreased by single treatment with HDACi and synergistically decreased by tBHP and HDACi. **C.** tBHP-induced expressions of *Cdkn2b* and* Cdkn2d* were increased in a dose-dependent manner. **D.** Expressions of *Cdkn2b* and* Cdkn2d* were increased by single treatment with HDACi and synergistically increased by cotreatment with tBHP and HDACi. **E.** Expressions of *Cdk4* and *6* were decreased by tBHP in a dose-dependent manner in Hep3B cells. **F.** Expressions of *Cdk4* and* 6* were decreased synergistically by co-treatment with tBHP and HDACi. **G.** Expressions of *Cdkn2b* and* Cdkn2d* were increased by tBHP dose-dependently. **H.** Expressions of *Cdkn2b* and* Cdk2d* were synergistically increased by co-treatment with tBHP and HDACi. Graphs are representative of the mean±SD from three independent experiments (*p<0.05, **p<0.01, vs. Vehicle). **I.** A representative blot of CDK1, CDK2, CDK4, CDK6, CDKN2B, and CDKN2D in HepG2 cells. Protein levels of CDK4 and 6 were decreased by tBHP whereas CDKN2B and CDKN2D were increased by tBHP in a dose-dependent manner. **J.** Protein levels of CDK1 and CDK2 were rarely changed by tBHP, HDACi, or combined treatment. Protein levels of CDK4 and 6 were dramatically decreased by combinatorial treatment with tBHP and HDACi. In contrast to CDK4 and 6, co-treatment with tBHP and HDACi increased the protein levels of CDKN2B and CDKN2D.

Quantitative PCR and western blots were conducted to validate MACE result. Expressions of CDKs such as *Cdk4* and *6* were investigated after oxidative stress induction with tBHP in HCC. *Cdk4* and *Cdk6* mRNA expressions were dose-dependently decreased by tBHP in HepG2 cells (Fig. 2A). Expressions of cell cycle regulators were investigated following treatment with a low concentration of tBHP alone (25 μmol/L), HDACi treatment using SAHA (1 μmol/L) and MS-275 (1 μmol/L), or co-treatment with tBHP and HDACi. Single treatment with a low concentration of tBHP or HDACi showed negligible effects on the expression of *Cdk4* and *6*. Co-treatment with tBHP and HDACi significantly decreased the expressions of *Cdk4* and *6* (Fig. 2B). Expressions of *Cdkn2b* and Cdkn*2d* were significantly increased at a high concentration of tBHP (50~ μmol/L) and minuscule changes at a relatively low level of tBHP (~25 μmol/L, Fig. 2C). CDKN expressions were not altered through tBHP (25 μmol/L) treatment alone. HDACi treatment alone induced incremental changes to *Cdkn2b* and *Cdkn2d* levels. Co-treatment with tBHP and HDACi drastically increased the *Cdkn2b* and Cdkn*2d* expressions (Fig. 2D). Similar expression patterns of *Cdk4/6* and *Cdkn2b/2d* by treatment with tBHP and HDACi were observed in the Hep3B cells. Expressions of *Cdk4* and* 6* were significantly decreased by tBHP at a relatively high concentration of tBHP (Fig. 2E). Combinatorial treatment with tBHP and HDACi significantly decreased the expression of *Cdk4* and* 6* (Fig. 2F). Expression of *Cdkn2b* and Cdkn*2d* was increased by a high concentration of tBHP (Fig. 2G) and combined treatment with a low concentration of tBHP (25 μmol/L) and HDACi (1 μmol/L, Fig. 2H) in Hep3B cells.

Protein levels of CDKs and CDKNs were confirmed by western blot in HepG2 cells. The protein level of CDK1 and 2 was negligibly changed by tBHP. Changes in CDK4 and 6 protein levels were negligible at low concentrations of tBHP (~25 μmol/L), but drastic reductions became evident from 50 μmol/L onwards. Conversely, dose-dependent increases in the expressions of CDKN2B and CDKN2D were observed (Fig. 2I). Single treatment with tBHP, SAHA, MS275, or combined treatment with tBHP and HDACi showed negligible impact on CDK1 and 2, whereas co-treatment with tBHP and HDACi dramatically reduced CDK4 and 6 protein levels. Contrastingly, protein levels of CDKNs such as CDKN2B and CDKN2D underwent drastic increases after co-treatment with tBHP and HDACi. Only CDKN2D levels were influenced by a single treatment with HDACi (Fig. 2J).

### HDACi sensitizes palbociclib-induced G2/M cell cycle arrest in HCC

**Figure 3:**
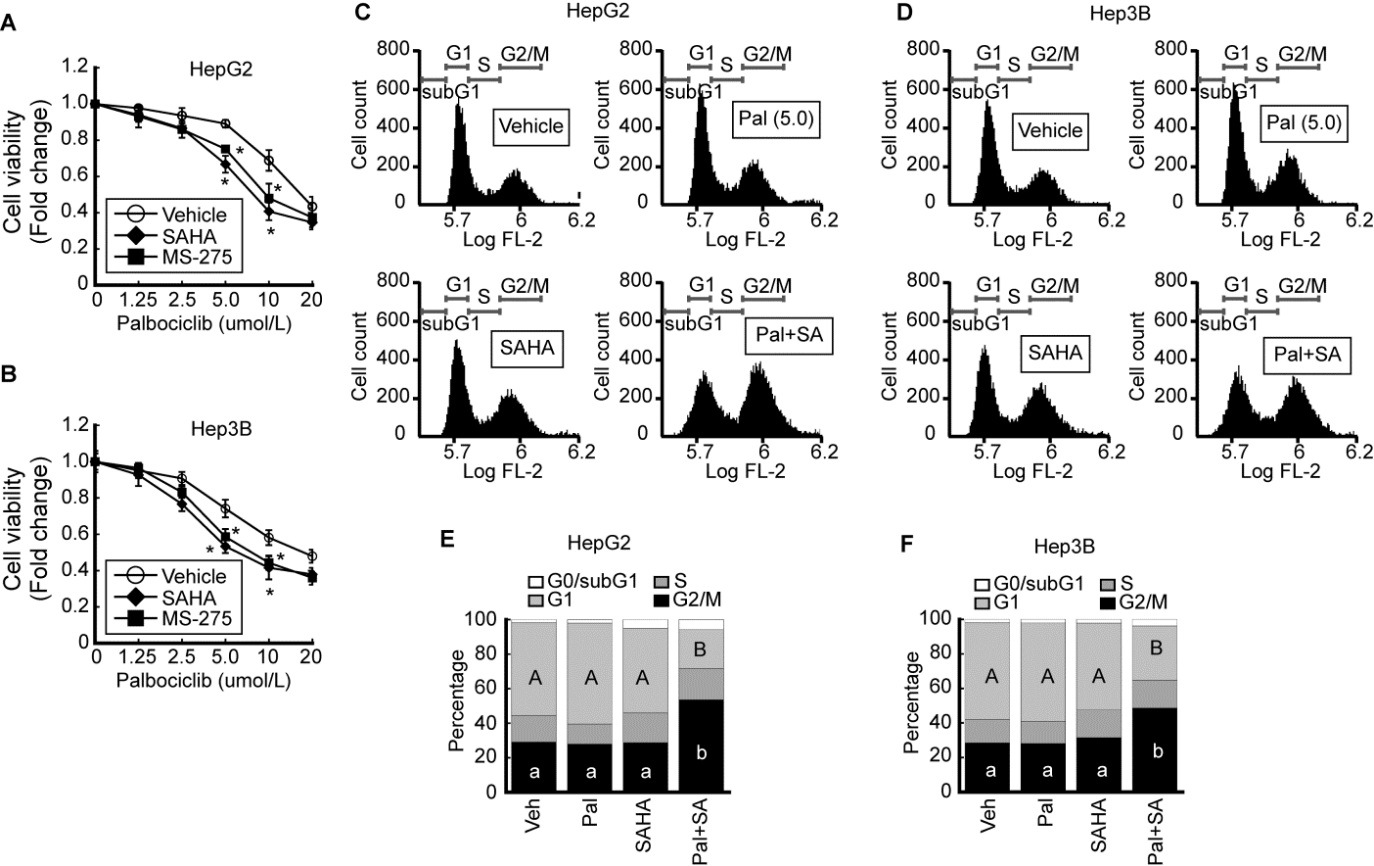
** HDACi increases palbociclib-induced G2/M cell cycle arrest in HCC.** Cell viabilities of HepG2 and Hep3B were analyzed after palbociclib or HDACi treatment. **A.** Cell viability of HepG2 was decreased by palbociclib, whose effect was enhanced by cotreatment with HDACi (1 µmol/L). **B.** Cell viability of Hep3B decreased by palbociclib, whose effect was increased by cotreatment with HDACi. Graphs show the mean±SD from three independent experiments (*p<0.05 vs. Vehicle). **C.** Single treatment with palbociclib (5 µmol/L) and SAHA (1 µmol/L) showed negligible effect on cell cycle of HepG2 cells. G2/M cell population was drastically increased by co-treatment with palbociclib and SAHA. **D.** FACS results of Hep3B showed decreased G1 and increased G2/M population by cotreatment with palbociclib and SAHA. Three independent FACS results of HepG2 **(E)** and Hep3B **(F)** were presented as stack columns. A one-way ANOVA followed by Dunnett's multiple comparison *post hoc* test was used for data analysis. Significant differences in G1 and G2/M cell cycle phases across all groups were denoted using an upper and lower case alphabet, respectively. Different alphabets indicate that the means between the pairs are significantly different (*p*<0.05).

We investigated the effect of palbociclib, a CDK4/6-specific inhibitor, on cell viability. The viability of HepG2 was decreased by palbociclib (IC_50_ = 34.9 ± 0.91 μmol/L) in a dose-dependent manner. Pairing palbociclib with either SAHA (IC_50_ = 25.0 ± 0.92 μmol/L) or MS-275 (IC_50_ = 27.5 ± 0.94 μmol/L) enhanced its effect on HepG2 cells viability (Fig. 3A). Similar trend was observed from Hep3B cells upon palbociclib treatment (IC_50_ = 33.8 ± 0.89 μmol/L), and incorporating SAHA (IC_50_ = 24.3 ± 0.72 μmol/L) or MS-275 (IC_50_ = 25.0 ± 0.9 μmol/L) incurred further viability reduction in a dose-dependent manner (Fig. 3B). To confirm that the decreased cell viability was caused by cell cycle arrest or cell death, we analyzed the palbociclib-induced changes to the cell cycle using FACS. Marginal increases in G1 cell populations were observed at relatively low palbociclib concentrations (5 μmol/L), or SAHA (1 μmol/L) whereas drastic G2/M cell cycle arrest in HepG2 cells became evident upon combined treatment with palbociclib and SAHA (Fig. 3C). Similar changes to the cell cycle were observed following palbociclib, SAHA, or combined treatment (Pal+SA) in Hep3B cells. Combined treatment with palbociclib and SAHA induced drastic G2/M cell cycle arrest in Hep3B (Fig. 3D). Results from three independent FACS analysis of HepG2 (Fig. 3E) and Hep3B (Fig. 3F) were presented as stack column.

### CDK4/6 inhibition decreases the activity of FOXM1

**Figure 4:**
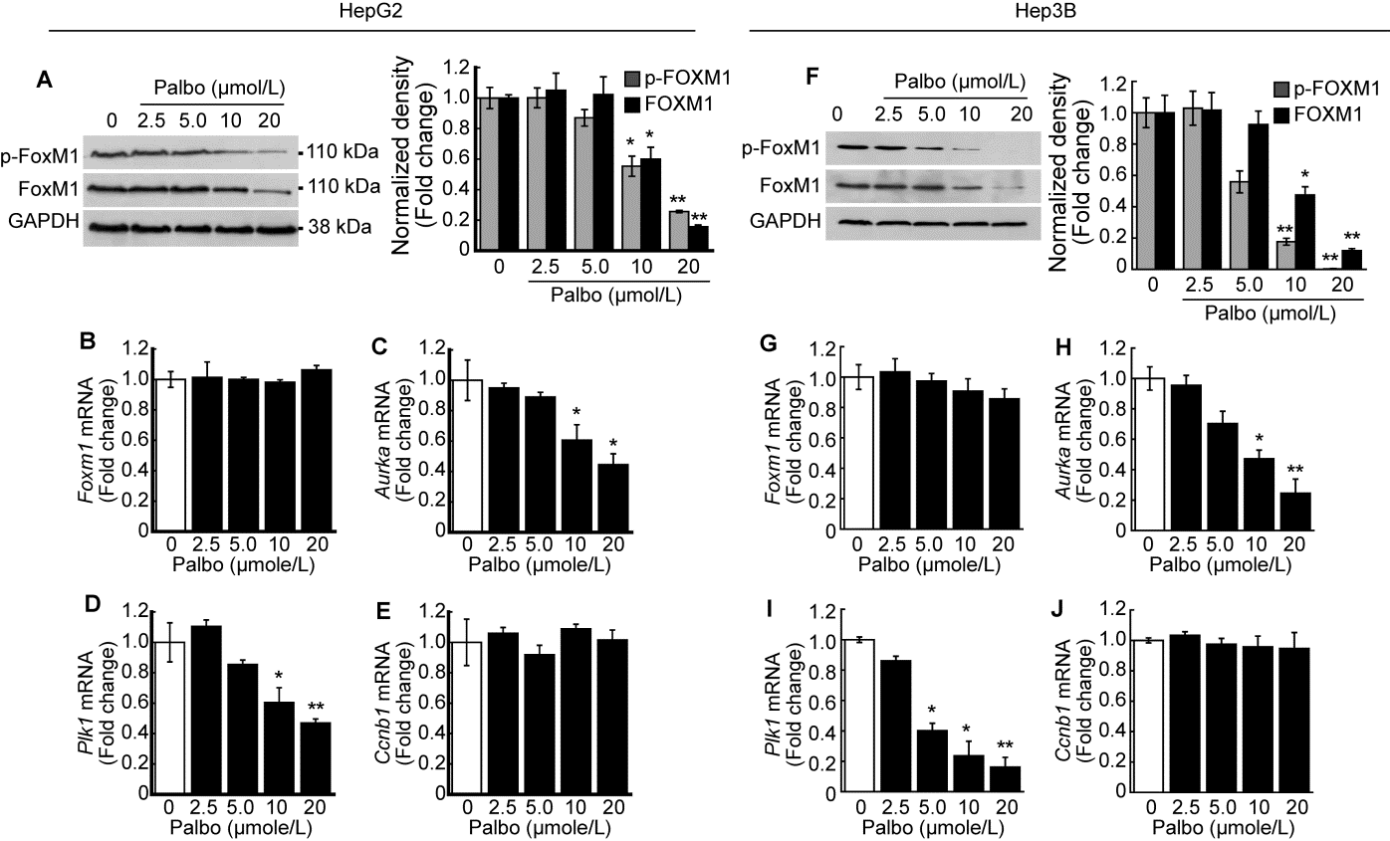
** Palbociclib suppresses FOXM1 activity in the HCC.** HepG2 cells were treated with the indicated concentration of palbociclib for 24 h. **A.** Representative blot of phospho-FOXM1 (pThr600), total FOXM1, and GAPDH. Densitometric analysis of three independent blots were performed. Palbociclib treatment decreased phosphorylated and total protein levels of FOXM1 in a dose-dependent manner. Expressions of *Foxm1* **(B)** and its target genes including *Aurka* **(C)**, *Plk1* **(D)**, and *Ccnb1* **(E)** were analyzed after treatment with palbociclib. Palbociclib treatment showed negligible effects on the expression of *Foxm1* and *Ccnb1*, while expressions of *Aurka* and *Plk1* were significantly decreased by palbociclib in a dose-dependent manner. **F.** Palbociclib reduced the phosphorylated and total protein levels of FOXM1 in Hep3B cells. **G.** Expressions of *Foxm1* mRNA were barely affected by palbociclib, whereas expressions of *Aurka* **(H)** and *Plk1* **(I)** mRNA were significantly decreased by palbociclib. **J.** Expression of *Ccnb1* mRNA was not affected by palbociclib up to 20 µmol/L. Data are representative of the mean±SD from three independent experiments (*p<0.05, **p<0.01, vs. Vehicle).

We investigated the phosphorylation level of FOXM1 after treatment with palbociclib. A dose-dependent decline in both phosphorylated and total FOXM1 expressions was observed upon palbociclib treatment in HepG2 cells (Fig. 4A). Contrary to this finding, no changes in *Foxm1* mRNA expressions were observed following palbociclib treatment (Fig. 4B). Next, we analyzed the expressions of well-known FOXM1 target genes post-treatment with palbociclib. Expression of FOXM1 target genes such as *Aurka* (Fig. 4C) and *Plk1* (Fig. 4D) was significantly decreased by palbociclib in a dose-dependent manner, although its effects on *Ccnb1* expression was negligible (Fig. 4E). Similar findings were observed following palbociclib treatment in Hep3B cells. Notably, both total and phospho-FOXM1 levels were decreased via palbociclib treatment in a dose-dependent manner (Fig. 4F). Expression of *Foxm1* mRNA was negligibly changed by palbociclib (Fig. 4G). Expression of FOXM1 target genes such as *Aurka* (Fig. 4H) and *Plk1* (Fig. 4I) was significantly decreased by palbociclib. Palbociclib showed negligible effect on the expression of *Ccnb1* (Fig. 4J).

### Double knockdown of CDKN2B (P15) and CDKN2D (P19) restored the FOXM1 activity suppressed by oxidative stress and HDACi

**Figure 5:**
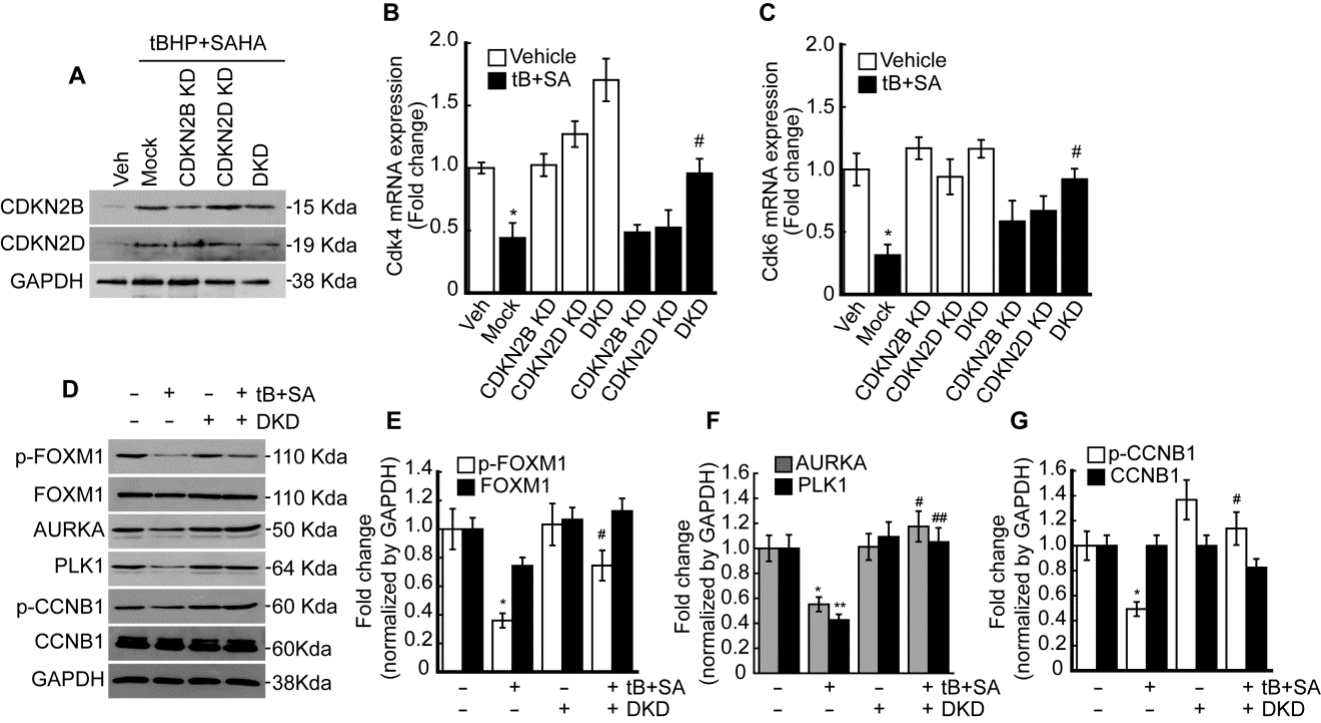
** Double knockdown of CDKN2B and CDKN2D reduces the effect of tBHP and SAHA.** Small interfering RNAs were transfected into the HepG2 cells for 24 h, which were subsequently incubated with tBHP and SAHA for CDKN2B and CDKN2D inductions. **A.** Western blot showed specific knockdown of CDKN2B and CDKN2D by siRNA. Co-treatment with tBHP and SAHA reduced the expressions of *Cdk4* **(B)** and *Cdk6* **(C)**, which were significantly restored by CDKN2B and CDKN2D double knockdown. Graphs are representative of the mean±SD from three independent experiments (*p<0.05, vs. Vehicle, ^#^*p*<0.05, vs. tBHP+SAHA). Representative blot images **(D)** and bands density analysis showed that double knockdown of CDKN2B and CDKN2D reversed the tBHP and SAHA-induced suppression of phospho-FOXM1 **(E)**, AURKA and PLK1 **(F)**, as well as phospho-CCNB1 **(G)**. Graphs are representative of the mean±SD from three independent experiments (*p<0.05, **p<0.01, vs. Vehicle, ^#^*p*<0.05, ^##^* p<*0.01 vs. tBHP+SAHA).

**Figure 6:**
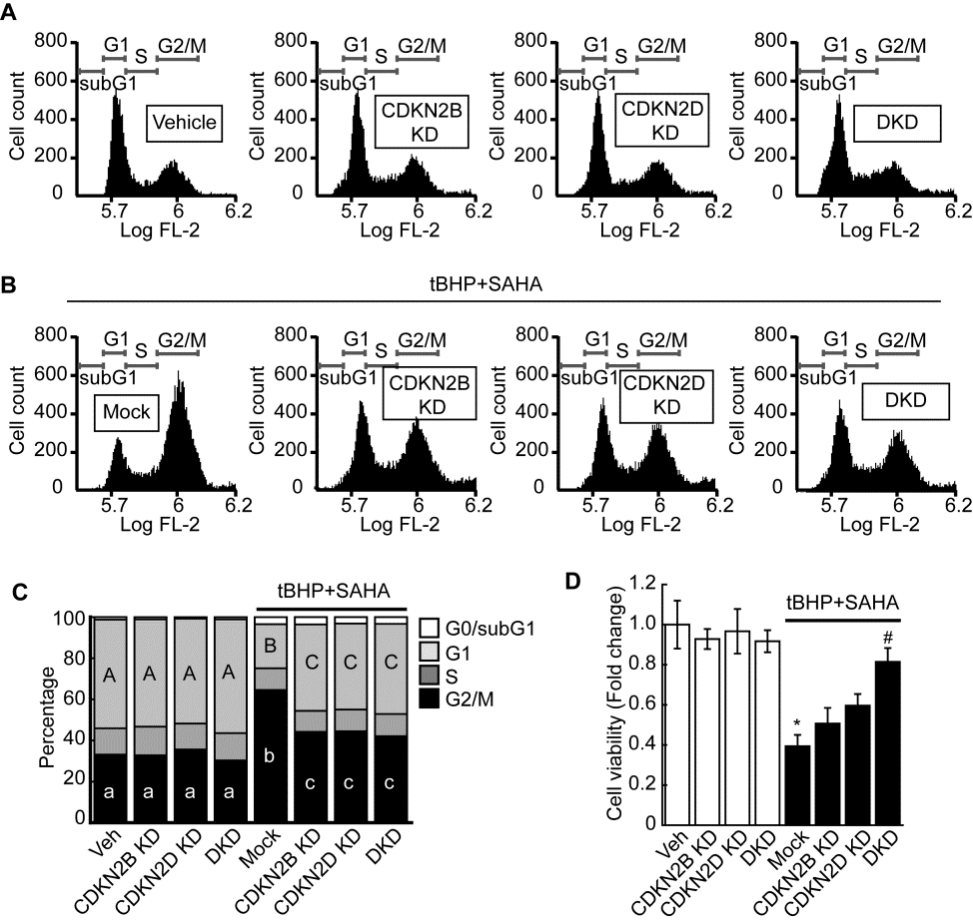
** Double knockdown of CDKN2B and CDKN2D reversed the G2/M cell cycle arrest and decreased cell viability of HepG2 cells induced by tBHP and SAHA. A.** Single knockdown or double knockdown demonstrated negligible effects on the HepG2 cell cycle. **B.** Single or double knockdown of CDKN2B and CDKN2D partially restored tBHP and SAHA-induced G2/M cell cycle arrest. **C.** Three independent FACS experiment results were summarized as stacked columns. A one-way ANOVA followed by Dunnett's multiple comparison *post hoc* test was used for data analysis. Significant differences in G1 and G2/M cell cycle phases across all groups were denoted using an upper and lower case alphabet, respectively. Different alphabets indicate that the means between the pairs are significantly different (*p*<0.05). **D.** Cell viability was investigated by using the CCK8 agent. Neither single nor double knockdown of CDKN2B and CDKN2D affected HCC cell viability. Double knockdown restored the HepG2 cell viability reduction induced through tBHP and SAHA treatment. Data are representative of the mean±SD from three independent experiments (*p<0.05, vs. Vehicle, ^#^*p*<0.05, vs. tBHP+SAHA).

Previous RNAseq and qPCR results revealed that combined treatment with tBHP and HDACi induced the expressions of specific CDK4/6 inhibitors CDKN2B and CDKN2D in HepG2 cells. Therefore, we investigated whether knockdown of these genes could restore the tBHP+HDACi-suppressed FOXM1 activity. Single or double knockdown of these genes in the HepG2 by siRNA transfection blocked the protein induction by tBHP+SAHA (Fig. 5A). Changes in *Cdk4/6* mRNA expression through single or double knockdown of CDKN2B and CDKN2D were negligible. However, double knockdown of CDKN2B and CDKN2D restored the expressions of *Cdk4* (Fig. 5B) and *Cdk6* (Fig. 5C) that were suppressed through tBHP and SAHA co-treatment. We then analyzed the effect of double knockdown on the signaling pathway which is critical for G2/M progression (Fig. 5D-G). Co-treatment with tBHP and SAHA decreased the phosphorylation level of FOXM1 (Fig. 5D and 5E), the protein levels of AURKA and PLK1 (Fig. 5D and 5F), and the phosphorylation level of CCNB1 (Fig. 5D and 5G), all of which were significantly restored by CDKN2B and CDKN2D double knockdown. Next, we tested the effect of CDKN2B, CDKN2D, or double knockdowns on the HepG2 cell cycle. Single or double knockdown of these genes showed a negligible effect on the cell cycle progression of HepG2 (Fig. 6A). Co-treatment with tBHP and SAHA induced drastic G2/M cell cycle arrest which was partially restored by knockdown of CDKN2B, CDKN2B, or both (Fig. 6B and 6C). Although cell viability of HepG2 was negligibly affected by the knockdowns, CDKN2B and CDKN2D double knockdown significantly reversed the cell viability reduction incurred by tBHP and SAHA co-treatment (Fig. 6D).

### HDACi and palbociclib induce proteosome-dependent FOXM1 degradation

**Figure 7:**
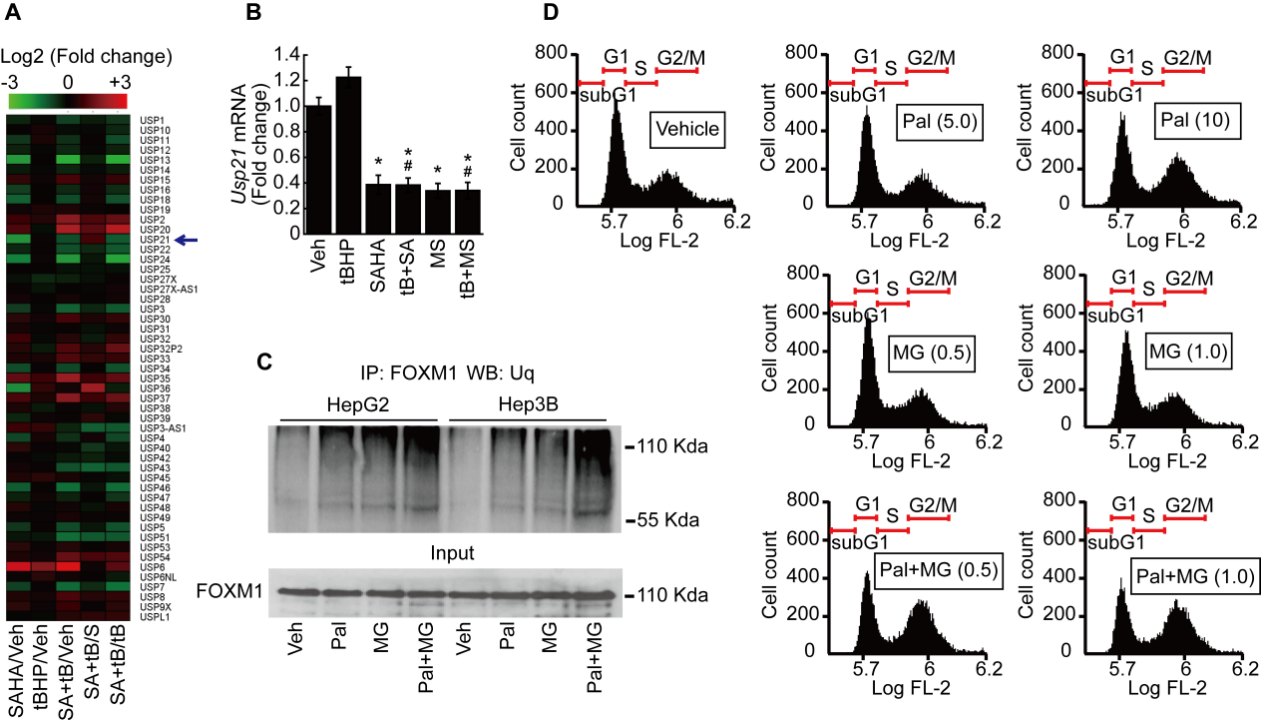
** Palbociclib induces proteasome-dependent FOXM1 degradation in HCC cells. A.** Heatmap of ubiquitin-specific peptidase showed that expression of *Usp21* was decreased by SAHA (1 µmol/L) treatment. Change in expression of *Usp21* by tBHP treatment was negligible in HepG2. **B.** Validation of MACE results by qPCR. SAHA and MS-275 (1 µmol/L) treatment significantly decreased the expression of *Usp21* in HepG2. Data are representative of the mean±SD from three independent experiments (*p<0.05, vs. Vehicle, #*p*<0.05, vs. tBHP). **C.** Ubiquitination level of FOXM1 was analyzed by immunoprecipitation with FOXM1 antibody followed by western blot with ubiquitin antibody. Ubiquitination level of FOXM1 was increased by palbociclib (10 µmol/L) and MG132 (1 µmol/L) compared to that of the vehicle control. Ubiquitination level of FOXM1 was dramatically increased by combined treatment with palbociclib and MG132. D. Effect of palbociclib and MG142 on cell cycle was analyzed by FACS. Low doses of MG132 (0.5 and 1 µmol/L) had negligible effects on the HepG2 cell cycle. MG132 treatment showed negligible effect on palbociclib (10 μmol/L)-induced G2/M cell cycle arrest in HepG2 cells.

It has been reported that ubiquitin-specific peptidases (USP) play a role in the maintenance of FOXM1 protein by preventing its proteasomal degradation 21. We analyzed the expression of USPs from our MACE results. USP21, which catalyzes the deubiquitination of FOXM1, was decreased by SAHA treatment (Fig. 7A). MACE result was validated by qPCR after HepG2 cells were treated with tBHP, SAHA, MS-275, or tBHP and HDACi combined. Low levels of oxidative stress induced by tBHP showed negligible effects on mRNA expression of *Usp21*. Both of the HDACi significantly suppressed the expression of *Usp21* (Fig. 7B). Immunoprecipitations were performed to confirm that palbociclib-induced FOXM1 degradation was the result of ubiquitination and proteasome-dependent pathway. Single treatment with palbociclib or MG132 alone increased the ubiquitination level of FOXM1. Combined treatment with palbociclib and MG132 drastically increased the ubiquitinated FOXM1 protein level in HepG2 and Hep3B cells (Fig. 7C). Low concentrations of MG132 had negligible effects on HepG2 cell cycle. MG132, regardless of concentration, did not revert the G2/M cell cycle arrest incurred by palbociclib (Fig. 7D).

### Phosphorylation is critical to the transcriptional activity of FOXM1

**Figure 8:**
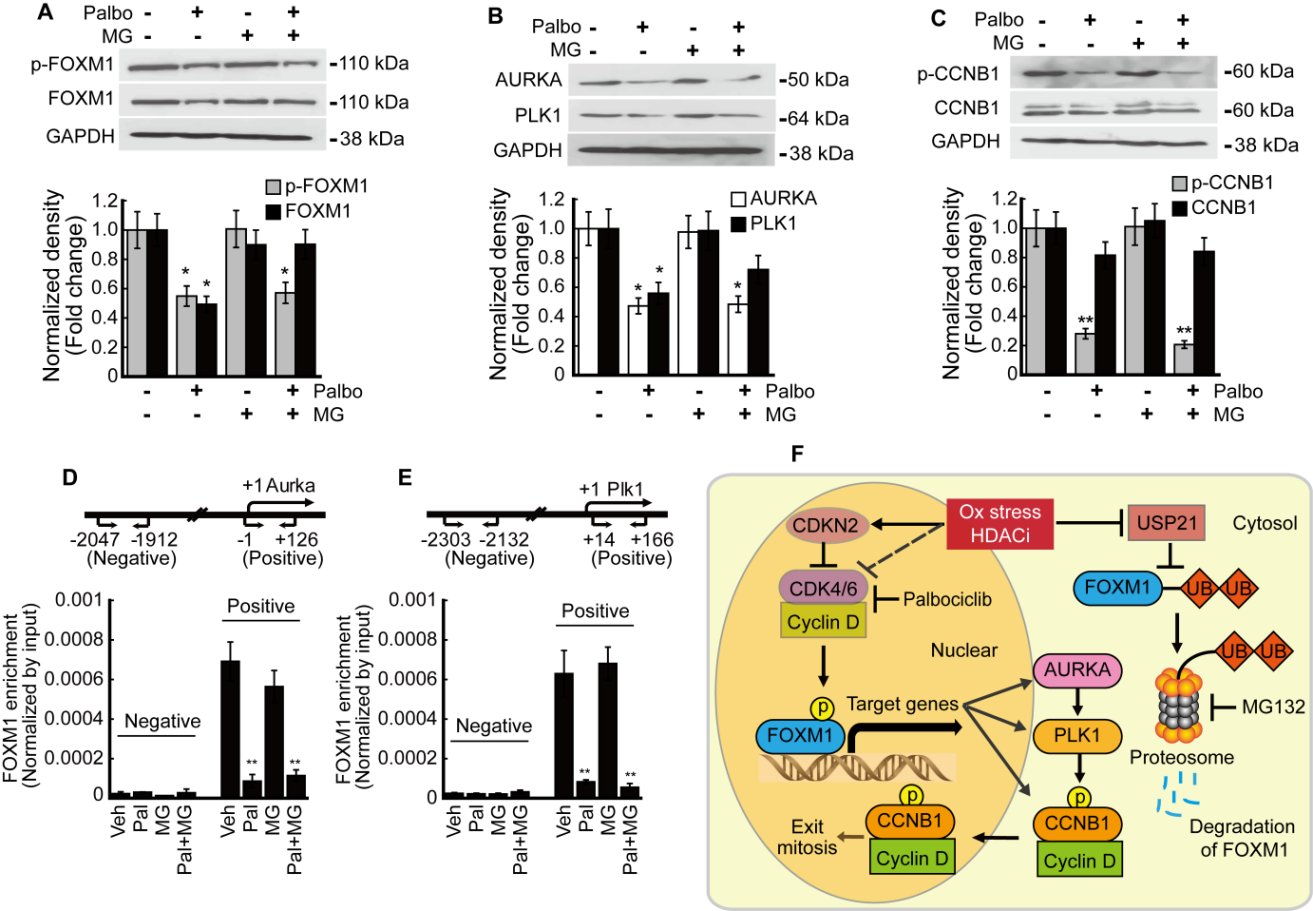
** Phosphorylation of FOXM1 is related to its transcriptional activity and the expression of target genes.** HepG2 cells were treated with palbociclib (10 µmol/L), MG132 (1 µmol/L), or co-treated with palbociclib and MG142 for 24 h. **A.** Protein levels of phospho- and total FOXM1 were significantly decreased by palbociclib treatment. Phosphorylation level of FOXM1 was not affected by MG132, but FOXM1 protein reduction by palbociclib was blocked by MG132. **B.** Expressions of FOXM1 target genes, AURKA and PLK1 were significantly decreased by palbociclib. MG132 did not revert AURKA and PLK1 inhibition by palbociclib. **C.** Protein level of phosphorylated CCNB1 was significantly decreased by palbociclib, but no changes to the total CCNB1 levels were observed from palbociclib, MG132, or combinatorial treatment. FOXM1 binding on the promoter regions of target genes *Aurka* **(D)** and *Plk1* **(E)** was analyzed by ChIP assay. Enrichment of FOXM1 was significantly decreased by palbociclib, which was negligibly affected by MG132. Graphs are representative of the mean±SD from three independent experiments (*p<0.05, **p<0.01, vs. Vehicle). **F.** Diagram summarizing the results of the present study. Oxidative stress and HDACi induce endogenous inhibitor of CDK4/6 (CDKN2 family) and suppress the expression of CDK4/6, which stabilize and activate FOXM1 by phosphorylation. HDACi suppresses the expression of USP21, which results in increased ubiquitination, and induced proteasomal degradation of FOXM1.

We analyzed the relationship between phosphorylation and transcriptional activity of FOXM1 in HepG2 cells. Palbociclib inhibited the expression of both phosphorylated and total FOXM1 forms. MG132 treatment blocked the palbociclib-induced FOXM1 degradation while phospho-FOXM1 remained decreased (Fig. 8A). FOXM1 target genes AURKA and PLK1 expressions were also inhibited by palbociclib, and MG132 treatment did not restore their expressions (Fig. 8B). Neither palbociclib, MG132, nor the two combined affected the total form CCNB1 expression. However, palbociclib reduced the amount of phospho-CCNB1 which was not reversed by MG132 (Fig. 8C). Enrichment of FOXM1 on the promoter of target genes including *Aurka* and *Plk1* was investigated by ChIP assay. FOXM1 enrichments on the promoters of *Aurka* (Fig. 8D) and *Plk1* (Fig. 6E) were significantly decreased by palbociclib, which was not reverted by MG132. Our findings were summarized as a schematic diagram in Fig. 8F.

## Discussion

The present study demonstrated that oxidative stress combined with HDACi suppressed CDK4/6 through CDKN2B and CDKN2D inductions, which decreased the stability and activity of FOXM1 by inhibiting its phosphorylation in the transactivating domain (TAD). Phosphorylation of FOXM1 in the TAD was closely related to the induction of target genes involved in G2/M regulation such as AURKA and PLK1. Additionally, our findings revealed that HDACi suppressed the expression of FOXM1 deubiquitinating peptidase *Usp21*, which resulted in proteasome-dependent proteolysis of FOXM1.

Massive analysis of cDNA ends (MACE) is an alternative variant of RNAseq, which requires a relatively short sequence (50-800 bp) from poly-A-tail of mRNA. In comparison to the standard RNAseq, MACE is a more powerful method for analyzing differential gene expressions and less susceptible to transcript length and degradation bias 22. We focused on changes to the cell cycle regulators because HCC cells that became sensitized to oxidative stress by HDACi underwent G2/M cell cycle arrest in our previous study 20. Fluctuating expressions of numerous cell cycle regulators were observed via oxidative stress and HDACi treatment. Interestingly, expressions of *Cdk4* and *Cdk6* were decreased while their endogenous inhibitors *Cdkn2b*, *Cdkn2d* were increased (Fig. 1). The role of CDKs and cyclins in mammalian cell cycle regulation has been well-reviewed elsewhere, which described a pivotal role of CDK4/6/Cyclin D in G1 phase, CDK2/cyclin E in G1/S transition, CDK1/cyclin A in G2 phase, and CDK1/Cyclin B in G2/M phase 23, 24. These cell cycle regulators are of particular importance for cancer studies because abnormal alterations to these genes were observed in most cancers. Somatic copy-number alteration analyses from more than 3000 specimens have revealed that *Ccnd1*, *Cdk4*, *Cdk6*, and *Ccne1* genes were listed in 30 of the most amplified genes associated with cancer, which highlight their potential as anti-cancer therapeutic targets 25, 26. Anti-cancer effects of the CDK4/6 inhibitors such as palbociclib, ribociclib, and abemaciclib in various types of cancers have been documented and these have all underwent phase III clinical trials 27-30. Since the canonical pathway of CDK4/6/cyclin D-RB-E2F in the G1 phase is well-defined, we initially anticipated CDK4/6 inhibitor-induced G1 cell cycle arrest or quiescence in our study. However, HCC cell lines such as HepG2 and Hep3B were arrested in G2/M phase by combinatorial treatment with palbociclib and HDACi (Fig. 3). Cell cycle progressions in some types of tumors, including embryonic carcinoma, choriocarcinoma, and Sertoli cell tumors were reported to be arrested in the G2/M phase in the presence of palbociclib or ribociclib 31. Thus, the mechanism enabling the bypassing of G1 checkpoint without CDK4/6 in these tumor cells including HCC, as well as the non-canonical function of CDK4/6 needs to be elucidated. Combined treatment with palbociclib and HDACi resulted in significantly increased G2/M cell cycle arrest (Fig. 3E and 3F) and decreased the cell viability of HCC (Fig. 3A and 3B). These results are similar to the previous studies reporting the synergistic effects of palbociclib and anti-cancer agents combined, which decreased cell viability and induced G2/M cell cycle arrest 32, 33.

Consistent with our RNAseq and qPCR results, HDACi such as butyrate and trichostatin A (TSA) specifically induced CDKN2B and CDKN2D in the cancer cells, including p21^WAF1/Cip^ deleted cancer cells 34, 35. CDKN2B and CDKN2D are included in the INK4 family (p15, p16, p18, and p19) which negatively regulate CDK4/6-cyclin D complex 36. Hence, we conducted knockdowns of CDKN2B and CDKN2D and then analyzed the mRNA expressions of *Cdk4* and *Cdk6*. Double knockdown of CDKN2B and CDKN2D reversed the *Cdk4* and *Cdk6* suppression incurred upon tBHP and SAHA co-treatment (Fig. 5B and 5C). The double knockdown also restored the FOXM1/AURKA/PLK1/CCNB1 signaling pathway, which is critical for G2/M cell cycle regulation (Fig. 5D-G). Cell cycle analysis revealed that both single and double knockdowns partially restored the G2/M cell cycle arrest (Fig. 6B), with the latter of the two almost fully reversing the cell viability reduction incurred by tBHP and SAHA treatment (Fig. 6D). While confirming the clinical relevance of these findings are necessary, acquiring HDACi-treated clinical HCC samples is extremely difficult since this is an uncommon clinical practice.

FOXM1 is known as a master regulator of proliferation, metastasis, and cell cycle in cancer cells 10. Our previous study has shown that FOXM1 inhibition by thiostrepton induced G2/M cell cycle arrest in HCC 20. Phosphorylation of FOXM1 TAD, especially at the sites Thr600, Thr611, and Thr638, are of critical importance since these enhance the transcriptional activity of FOXM1 and contribute to mitotic progression 37-39. We investigated both phosphorylated and total forms of FOXM1 because palbociclib treatment induced G2/M cell cycle arrest in the present study. Palbociclib treatment suppressed the expression of both phosphorylated and total forms of FOXM1 at a relatively high concentration (10 μmol/L~) without altering their mRNA expressions (Fig. 4A, 4B, 4F, and 4G). From these results, we speculated that CDK4/6 regulates the stability of FOXM1 protein rather than its mRNA transcription. Expressions of FOXM1 target genes such as *Aurka* and *Plk1* that play a critical role in G2/M progression were inhibited by palbociclib treatment (Fig. 4C, 4D, 4H, and 4I), except for *Ccnb1* (Fig. 4E and 4J). To assess the transcriptional activity of FOXM1, enrichment of FOXM1 in the promoters of *Aurka* and *Plk1* was investigated by ChIP assay. Palbociclib treatment significantly decreased the enrichment of FOXM1 near the transcription start site of *Aurka* (Fig. 8D) and *Plk1* (Fig. 8E). Interestingly, decreased FOXM1 protein level was reversed by MG132 treatment but reduced transcriptional activity of FOXM1 by palbociclib was not recovered (Fig. 8). Stability of the FOXM1 protein level was increased by USP21, a deubiquitination peptidase of FOXM1 21. Results of MACE and qPCR showed that HDACi treatment decreased the expression of *Usp21* (Fig. 7A and 7B). Since palbociclib treatment decreased the stability of FOXM1, we investigated the ubiquitin-dependent proteasomal degradation of FOXM1. Palbociclib treatment increased the ubiquitination level of FOXM1, which was further enhanced by MG132 (Fig. 7C). Although MG132 treatment inhibited FOXM1 degradation, neither palbociclib-inhibited FOXM1 binding affinity to its target gene promoters (Fig. 8D and 8E) nor the expressions of *Aurka* and *Plk1* (Fig. 8B) were restored via MG132 treatment. CCNB1 phosphorylation at Ser147 by PLK1 has been reported to be critical for progression into the M phase 40. Although insignificant changes were induced to the mRNA and protein expressions of total form CCNB1, palbociclib treatment inhibited PLK1 (Fig. 8B) and subsequently affected phosphorylated CCNB1 levels (Fig. 8C). Interestingly, although palbociclib-induced FOXM1 degradation was blocked by MG132 (Fig. 8A), it did not revert the G2/M cell cycle arrest induced by palbociclib treatment (Fig. 7D). From these results, we concluded that phosphorylation by CDK4/6 on TAD of FOXM1 is closely related to its transcriptional activity and stability.

The ROS-scavenging capacity of cancer cells is higher than that of normal cells because cancer cells are exposed to a high level of ROS resulting from genetic, metabolic, and microenvironment-associated alterations 41. In many types of cancers, high capacity of ROS detoxification is related to multi-drug resistance 5. Thus, many studies have focused on modulating the redox status of cancer as a potential therapeutic strategy 41. In line with this rationale, several anticancer drugs such as topotecan, doxorubicin, etoposide, and procarbazine are currently used in clinical settings for cancer treatment. These drugs, once metabolized by the tumor cells, produce vast quantities of ROS that promote the oxidation of intracellular macromolecules such as lipid, protein, and nucleic acids 8. However, increased ROS levels by anticancer drugs cause diverse side effects even in normal cells. One potential strategy that addresses this issue is combining a low concentration of ROS-producing anticancer drug with an agent that sufficiently dampens the oxidative stress threshold in cancer cells. Resultantly, combinatorial therapy involving anticancer drugs and HDACi has emerged as a cancer treatment option 19. Our previous study has shown that HDACi sensitizes HCC to oxidative stress induced by tBHP, which resulted in G2/M cell cycle arrest at a low level of oxidative stress 20. Therefore, we analyzed the expression of cell cycle regulators such as CDKs and CDKNs after treatment with SAHA and/or tBHP in the HCC. MACE result showed that CDK4 and 6 were decreased and CDKN2B and CDKN2D were increased by SAHA (Fig. 1). Cdkn1a (P21) expression was increased by combined treatment with tBHP and HDACi whereas its effect was reversed in the case of Tp53, a well-known upstream protein of p21 (Fig. 1). From these results, we speculated that G2/M cell cycle arrest induced by HDACi and oxidative stress is caused through P53-independent pathway. Cell cycle arrest and apoptosis resulting from DNA damage/P53/P21 pathway is well-established 42 and this is also applicable to the P53-independent activation of P21 and its signaling pathway 43.

FOXM1 acts as a master regulator of G2/M cell cycle as well as a critical regulator of oxidative stress during oncogenesis. ROS scavenging enzymes such as MnSOD, catalase, and PRDX3 are induced by FOXM1, which contributes to reducing intracellular ROS levels. Thus, FOXM1 activity is essential to cancer cells expressing ROS-producing oncogenes such as RAS or AKT 44. Therefore, FOXM1 is a potential target for cancer therapy. HDACi treatment not only reduced the expression of Cdk4 and 6, but also increased expressions of their endogenous inhibitor, CDKN2B and CDKN2D as well. Knockdown of CDKN2B and CDKN2D reversed decreased phosphorylation level of FOXM1 caused by co-treatment with tBHP and SAHA (Fig. 5D). Additionally, HDACi suppressed Usp21, a deubiquitinating enzyme of FOXM1, which resulted in increased ubiquitination on FOXM1 and its degradation through the proteasome-dependent pathway (Fig. 7A-C). These results indicate that HDACi is a FOXM1 targeting agent indirectly.

In conclusion, HDACi induced the expression of CDKN2B and CDKN2D, which resulted in decreased activity of CDK4/6 as well as suppression of Usp21. These results were related to diminished activity and stability of FOXM1, which plays a critical role in cell cycle progression and anti-oxidative activity in HCC. Thus, HDACi combination with other anticancer agents could be a potential treatment option for cancer cells expressing FOXM1.

## Supplementary Material

Supplementary table.
