# Supplementary material for: Histone Deacetylase Inhibitor-Induced CDKN2B and CDKN2D Contribute to G2/M Cell Cycle Arrest Incurred by Oxidative Stress in Hepatocellular Carcinoma Cells via Forkhead Box M1 Suppression
Source: J Cancer. 2021 Jun 22;12(17):5086–98. doi: 10.7150/jca.60027 (PMC8317537; doi:10.7150/jca.60027)
Supplement: Supplementary file 1 — Supplementary table. [file jcav12p5086s1.pdf]

Supplementary Material

Table S1. Primer sequences used in the present study

Primer set for qPCR			
Genes	Sequence (5’ to 3’)	Tm (°C)	Amplicon size (bp)
Cdk4	F: TAACTGGAGATGGCTGTGGG<br>R: TATCCTTCTCTGTGGGTGGC	60	158
Cdk6	F: CCAGGCAGGCTTTTCATTCA<br>R: AAGTATGGGTGAGACAGGGC	60	151
Cdkn2b	F: GAATGCGCGAGGAGAACAAG<br>R: TCATCATGACCTGGATCGCG	60	168
Cdkn2d	F: GTTGGACAGAGAAGGGCTCC<br>R: CGTGCACACTTCAGGTCTCT	60	186
Foxm1	F: ACAAGTGGATCTGCTTGCCA<br>R: CACCCACACTCTGCTTCAGT	60	169
Aurka	F: GCCAAGCCTGGTAAAGCTGT<br>R: AAACCCAATCAGGCCTACCG	60	151
Plk1	F: ACATACCGCCTGAGTCTCCT<br>R: CAGATGCAGGTGGGAGTGAG	60	183
Ccnb1	F: ACTTTGGGAGGCAGAGTTGG<br>R: GATTCTCCTGCCTCAGCCTC	60	160
USP21	F: GTATGCCCTTTGCAACCACT<br>R: GATGCCACCTGGTTTTCACT	60	129
Gapdh	F: GGGAGCCAAAAGGGTCATCA<br>R: GGCATGGACTGTGGTCATGA	60	199
Primer set for ChIP			
Aurka (Positive)	F: GTGGCCCACCCCTAACTTCT<br>R: GTCCTCGTGTGCTCACCTGC	60	
Aurka (Negative)	F: AAAGTGCTGGGATTACGGGC<br>R: TTAGCATGACGGTATCTGGCATG	60	
Plk1 (Positive)	F: GGGCGGGTTTGGATTTTA<br>R: AGTCACTGCAGCACTCATGC	60	
Plk1 (Negative)	F: AATCGCTTGAACCCAGGAGG<br>R: AGGGGTCCTGGACTTAACCA	60	
